# Supplementary material for: Dynamic transcriptomic profiles of zebrafish gills in response to zinc supplementation
Source: BMC Genomics. 2010 Oct 11;11:553. doi: 10.1186/1471-2164-11-553 (PMC3091702; doi:10.1186/1471-2164-11-553)
Supplement: Additional file 2 — Interactive Direct Interaction Network representing the molecular interactions between zinc, copper, iron, calcium and proteins encoded by transcripts changed by zinc supplementation. Mini web-site containing index.html and hyperlinked pages in subdirectory describing a Direct Interaction Network automatically generated based on curated interactions contained within the proprietary PathwayArchitect database. Ovals represent proteins and the circles symbolize metal ions. Objects are coloured by their abundance in zebrafish at the time-point they were significantly different from the control is a scale from -4 fold (dark green) to +4 fold (dark red). Where significant differences were found at more than one time-point, the colour overlay shows expression at the first instance. Dark blue squares denote 'binding', and light blue squares 'expression'; green squares stand for 'regulation', green diamonds for 'metabolism', and green circles for 'promoter binding'. Arrow heads indicate directionality of the interaction where annotated. All nodes and edges can be further interrogated by selecting the relative area of the image. [file 1471-2164-11-553-S2.zip › PathwayArchitect Zn xs DIN/523108.html]

# BINDING:

|  |  |
| --- | --- |
| Type | BINDING |
| Effect | None |


---

|  |  |
| --- | --- |
| Score | 0 |


---

|  |  |
| --- | --- |
| Reference Count | 6 |


---

|  |  |
| --- | --- |
| Mechanism | Unknown |


---

|  |  |
| --- | --- |
| Reference:0 || Sentence | "Some interactions involve recognition of continuous epitopes, where there is no necessity for a tertiary structure and very often the binding conformation is induced during the process of interaction, for example phosducin binding to the betagamma subunits (Gtbetagamma) of the heterotrimeric G protein transducin." |
| PMID | 11076027 |
| Year | 2000 |
| Species | Human |
| Journal | Biol Chem |
| RefScore | 3 |
| Source | PArchNLP |
  |
|  |  |


---

|  |  |
| --- | --- |
 Reference:1 || Sentence | "Phosducin has been shown to bind the beta, gamma subunits of the retinal G-protein transducin." |
| PMID | 2071146 |
| Year | 1991 |
| Species | Mouse |
|  | Rat |
| Journal | Genomics |
| RefScore | 1 |
| Source | PArchNLP |
  ||  |  |


---

|  |  |
| --- | --- |
 Reference:2 || Sentence | "Phosducin (Pd), a principal protein of retinal photoreceptor cells, modulates the phototransduction cascade by interacting with transducin." |
| PMID | 8224906 |
| Year | 1993 |
| Species | Human |
|  | Mouse |
| Journal | Gene |
| RefScore | 1 |
| Source | PArchNLP |
  ||  |  |


---

|  |  |
| --- | --- |
 Reference:3 || Sentence | "Phosducin, a principal protein of the retinal photoreceptor cells, modulates the phototransduction cascade by interacting with transducin." |
| PMID | 8188267 |
| Year | 1994 |
| Species | Human |
|  | Mouse |
| Journal | Genomics |
| RefScore | 1 |
| Source | PArchNLP |
  ||  |  |


---

|  |  |
| --- | --- |
 Reference:4 || Sentence | First, we have demonstrated that transducin beta gamma subunits interact with phosducin along their entire intracellular translocation route, as evident from their co-precipitation in serial tangential sections from light-adapted but not dark-adapted retinas. |
| Year | 2004 |
| PMID | 14973130 |
| Species | Mouse |
|  | Rat |
| Journal | J Biol Chem |
| RefScore | 0 |
| Source | PArchNLP |
  ||  |  |


---

|  |  |
| --- | --- |
 Reference:5 || Sentence | Together, these data indicate that phosducin binding to transducin beta gamma subunits facilitates transducin translocation. |
| Year | 2004 |
| PMID | 14973130 |
| Species | Mouse |
|  | Rat |
| Journal | J Biol Chem |
| RefScore | 1 |
| Source | PArchNLP |
  |


---

|  |  |
| --- | --- |
